# Pressor recovery after acute stress is impaired in high fructose‐fed Lean Zucker rats

**DOI:** 10.14814/phy2.12758

**Published:** 2016-06-22

**Authors:** Jennifer A. Thompson, Gerard D'Angelo, James D. Mintz, David J. Fulton, David W. Stepp

**Affiliations:** ^1^ Vascular Biology CenterGeorgia Regents UniversityAugustaGeorgia

**Keywords:** Fructose, insulin resistance, pressor responses, stress, *β*‐adrenergic

## Abstract

Insulin resistance is a powerful predictor of cardiovascular disease; however, the mechanistic link remains unclear. This study aims to determine if early cardiovascular changes associated with short‐term fructose feeding in the absence of obesity manifest as abnormal blood pressure control. Metabolic dysfunction was induced in Lean Zucker rats by short‐term high‐fructose feeding. Rats were implanted with telemetry devices for the measurement of mean arterial blood pressure (MAP) and subjected to air jet stress at 5 and 8 weeks after feeding. Additional animals were catheterized under anesthesia for the determination of MAP and blood flow responses in the hind limb and mesenteric vascular beds to intravenous injection of isoproterenol (0.001–0.5 *μ*m), a *β*‐adrenergic agonist. Metabolic dysfunction in high‐fructose rats was not accompanied by changes in 24‐h MAP. Yet, animals fed a high‐fructose diet for 8 weeks exhibited a marked impairment in blood pressure recovery after air‐jet stress. Dose‐dependent decreases in MAP and peripheral blood flow in response to isoproterenol treatment were significantly attenuated in high‐fructose rats. These data suggest that impaired blood pressure recovery to acute mental stress precedes the onset of hypertension in the early stages of insulin resistance. Further, blunted responses to isoproterenol implicate *β*_2_‐adrenergic sensitivity as a possible mechanism responsible for altered blood pressure control after short‐term high‐fructose feeding.

## Introduction

The Metabolic syndrome (MetS), characterized as a constellation of risk factors including obesity, insulin resistance, and hypertension, is considered a major driver of cardiovascular disease (CVD) in Western society (Isomaa et al. 2001; Straznicky et al. 2014) The persistently high risk of death from cardiovascular complications in MetS despite current therapies (Wilson et al. 2005; Fruchart et al. 2014) warrants a better understanding of the mechanisms that link components of MetS to cardiovascular pathology.

Hypertension is a strong predictor of cardiovascular mortality and common feature of MetS, occurring in over 80% of patients (Mathews et al. 2003). Insulin resistance has been implicated as a principal causative factor in the development of hypertension in MetS. This supposition is reinforced by a sizeable body of literature reporting an increase in arterial blood pressure in rodents fed a high‐fructose diet, a model of insulin resistance in the absence of obesity (Dai and McNeill 1995; Cosenzi et al. 1999; Verma et al. 1999; Galipeau et al. 2001; Katovich et al. 2001; Catena et al. 2003; Hsieh 2004). Common among these studies is the use of tail‐cuff plethysmography for the measurement of arterial blood pressure. Our laboratory reproduced this reported increase in tail‐cuff values of arterial blood pressure, yet in the same study, failed to observe an increase in blood pressure when assessed with radiotelemetry, the gold standard in blood pressure measurement (D'Angelo et al. 2005). Radiotelemetry allows prolonged measurement of diurnal blood pressure and circumvents limitations inherent in the conventional tail‐cuff method such as mental stress due to physical restraint, exclusion of diastolic blood pressure, and variations in vasodilator capacity of the tail circulation. Thus, the aforementioned evidence purported to corroborate a link between insulin resistance and hypertension may in fact reflect a heightened pressor response to mental stress in high fructose‐fed rodents similar to “white‐coat hypertension” seen in humans. We therefore aimed to test the hypothesis that fructose feeding amplifies cardiovascular reactivity to acute mental stress.

## Materials and Methods

All experimental protocols were approved by the Institutional Animal Care and Use Committee of Georgia Regents University and conducted in accordance with the National Institutes of Health Guide for the Care and Use of Laboratory Animals.

### Animal model

Male lean Zucker rats (LZRs) were purchased from Harlan Laboratories (Indianapolis, IN) and housed in the animal care facility at Georgia Regents University, which is approved by the American Association for the Accreditation of Laboratory Animal Care. Zucker rats were selected to provide strain consistency with previous studies on metabolic disease and responses to stress (D'Angelo et al. 2006). Animals were maintained on a 12‐h light–dark cycle with access to food and water *ad libitium*. After 1 week of acclimatization, some animals underwent surgery for telemetry placement. Animals were randomly assigned to a high‐fructose diet (Fructose diet formula TD pellets 89,247; % total kcal: 66% fructose, 22% casein, 12% lard,) or control diet (Standard rat chow Harlan Teklad Laboratories 8604). Sodium consumption for control and fructose diets averaged 72 ± 2 and 110 ± 5 mg/day, respectively. At 5 and 8 weeks of feeding, animals implanted with telemetry devices were subjected to air jet stress. A separate set of animals was assessed for hemodynamic responses to *β*‐adrenergic stimulation at 8 weeks of high‐fructose feeding. Animals were killed by decapitation.

### Metabolic parameters

After 8 weeks of feeding, trunk blood was collected, fasting blood glucose was evaluated using a glucometer (Abbott Laboratories, Bedford MA) and plasma frozen for analysis. Colorimetric assays (Wako Diagnostics, Mountain View, CA) were used to measure plasma levels of total cholesterol and triglycerides. Plasma insulin was measured with the Mercodia Rat Insulin ELISA assay (ALPCO Diagnostics, Salem NH).

### Arterial pressure

Telemetry transmitters (Data Sciences International, St. Paul, MN) were implanted in anesthetized rats (ketamine/xylazine, 50 mg/kg IP) according to the manufacturer's instructions. Briefly, a midline incision was made to expose the abdominal aorta. The catheter was inserted proximal to the iliac bifurcation and the transmitter body secured to the abdominal wall with suture and VetBond adhesive. After surgery, animals were housed in individual cages and recovered over a 7‐day period in a room separate from that used for air‐jet stress experiments. Mean arterial blood pressure (MAP) and heart rate (HR) were continuously recorded using Dataquest A.R. T. (Data Sciences International, St. Paul MN).

### Air‐jet stress

Air‐jet stress was used to elicit increases in blood pressure to acute stress. Pressor reactivity was assessed in rats fed with a high‐fructose or control diet for 5 and 8 weeks (*n* = 7), as described previously (D'Angelo et al. 2006). Animals were transferred to a sound‐proofed room and allowed at least a 15‐min acclimatization period during which physical activity, MAP, and HR stabilized. Animals were then placed in aerated plexiglass restrainers and monitored for at least 15 min. Once stable arterial pressures and HRs were achieved, air‐jet stress was applied. Compressed air (15 lb/in^2^) was aimed at the forehead through an opening at the front of the restrainer, delivered in 2‐sec pulses administered every 10 sec over a 3‐min period. Following air‐jet stress, MAP and HR were recorded for an additional 20 min while the animal remained in the restrainer. Rats were returned to the holding room after the 20‐min recovery.

### Regional blood flow

After 8 weeks of high‐fructose feeding, blood flow responses to isoproterenol in the hind limb and mesentery were evaluated using a Transonic T402 Flowmeter, as previously described (D'Angelo et al. 2006). Under isofluorane anesthesia (4–5% with 2 L/min O_2_ for induction, followed by 2.5–3% with 1 L/min for maintenance), the carotid artery and jugular vein were catheterized for measurement of MAP and drug delivery, respectively. A midline incision was made and the distal aorta at the iliac bifurcation or the superior mesenteric artery was exposed. The adventitial tissue was gently removed, Transonic flow probes (IPRB for renal and mesenteric; 2PS for aorta) placed around the vessel and a coating of HR conductance jelly applied. Randomized boluses of isoproterenol (0.001–0.5 *μ*mol) were administered intravenously. MAP and blood flow were recorded and conductance calculated as flow divided by pressure.

### Statistical analysis

Baseline MAP and HR values are reported as 24‐h means. Total pressor response to air‐jet stress and poststress recovery were calculated with the equation: Σ[(pressure–pre‐air jet baseline) × 0.083)], where pressure is each measurement recorded during the delivery of air‐jet stress and the 20‐min poststress period; the pre‐air jet baseline is the average pressure during the 3 min preceding the onset of air pulses and 0.083 is the 5‐sec data collection interval. Data are expressed as area under the curve [(AUC) mmHg × minutes). Differences in AUC between the two groups were assessed with one‐way ANOVA and a Bonferroni post hoc test. Conductance was calculated as blood flow (mL/min/g) divided by MAP. Isoproterenol responses were analyzed by two‐way ANOVA with dose and diet as sources of variation. All baseline metabolic and cardiovascular parameters were compared by unpaired *t*‐test. Peak responses were compared using a *t*‐test. Statistical analyses were performed with Graph pad (Prism 5). Differences are considered significant at *P* < 0.05, and data expressed as mean ± SEM.

## Results

### Baseline characteristics

**Figure 1:**
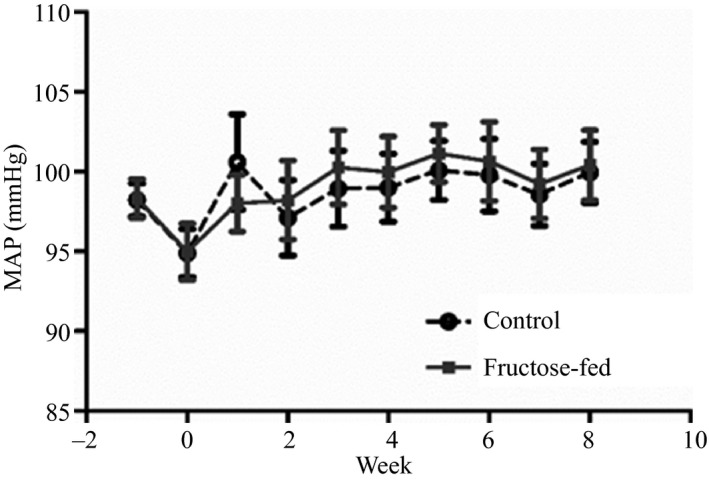
24‐h MAP calculated from telemetry readings in control rats and rats fed a high‐fructose diet for 8 weeks.

Baseline metabolic parameters are shown in Table 1. After 8 weeks of high‐fructose feeding, there was a 1.6‐fold increase in fasting blood glucose levels (*P* < 0.001), a fivefold increase in plasma insulin (*P* < 0.01), a threefold increase in triglyceride levels (*P* < 0.001) and cholesterol was increased by 1.5‐fold (*P* < 0.01). Body weight was not different between control and fructose‐fed groups. Metabolic dysfunction induced by 8 weeks of high‐fructose feeding was not accompanied by a change in 24‐h MAP in rats implanted with telemetry devices (Fig. 1). This lack of increase is not due to a deficit in sodium in the diet as fructose feeding was associated with a mildly higher, not lower, sodium intake.

**Table 1:** Baseline metabolic parameters

Parameter	Control	Fructose‐fed
Age (weeks)	12–18	12–18
Body weight (g)	347 ± 16.4	366 ± 8.5
Fasting blood glucose (mg/dL)	188 ± 12.8	306 ± 14.9^b^
Plasma insulin (*μ*g/mL)	0.18 ± 0.486	0.96 ± 0.272^a^
Serum triglyceride (mg/dL)	68.3 ± 8.87	200.9 ± 25.90^b^
Serum cholesterol (mg/dL)	50.0 ± 2.14	74.3 ± 3.86^a^

a *P < *0.01 versus Control.

b *P < *0.001 versus Control.

### Pressor responses to acute stress

**Figure 2:**
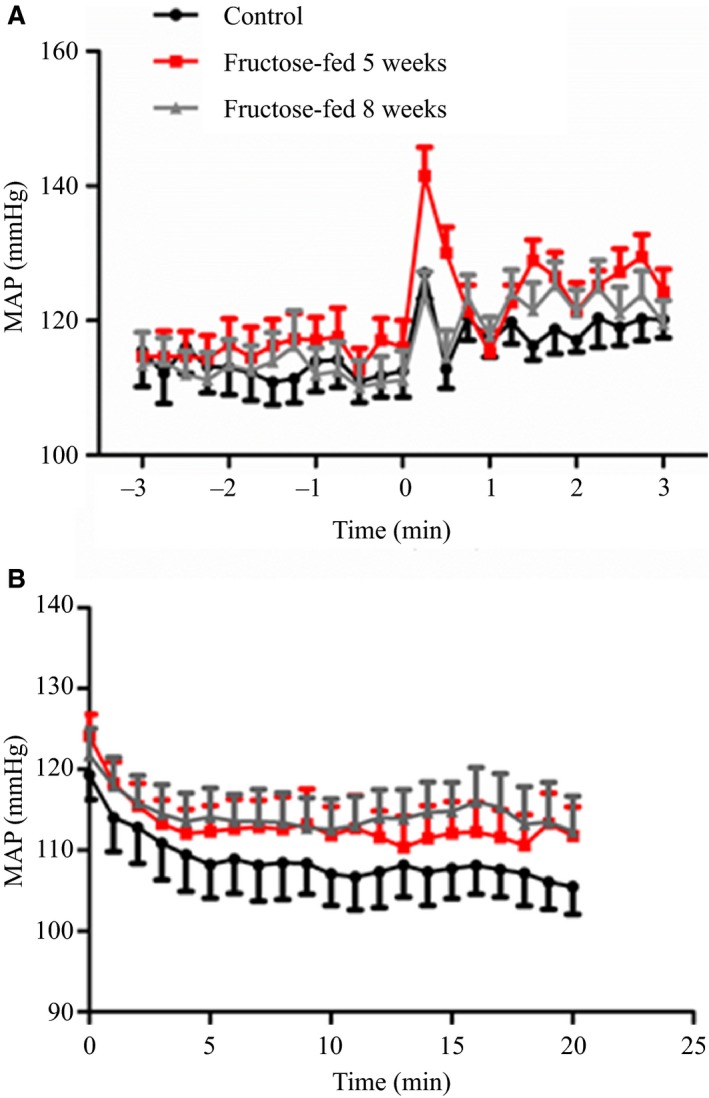
Blood pressure responses to air‐jet stress in control rats and rats fed a high‐fructose diet for 5 and 8 weeks. MAP was continuously monitored during the 3‐min pre‐stress period and the 3‐min stress period (A). Monitoring of MAP continues for 20‐min after cessation of air‐jet stress (B). *n* = 7

**Figure 3:**
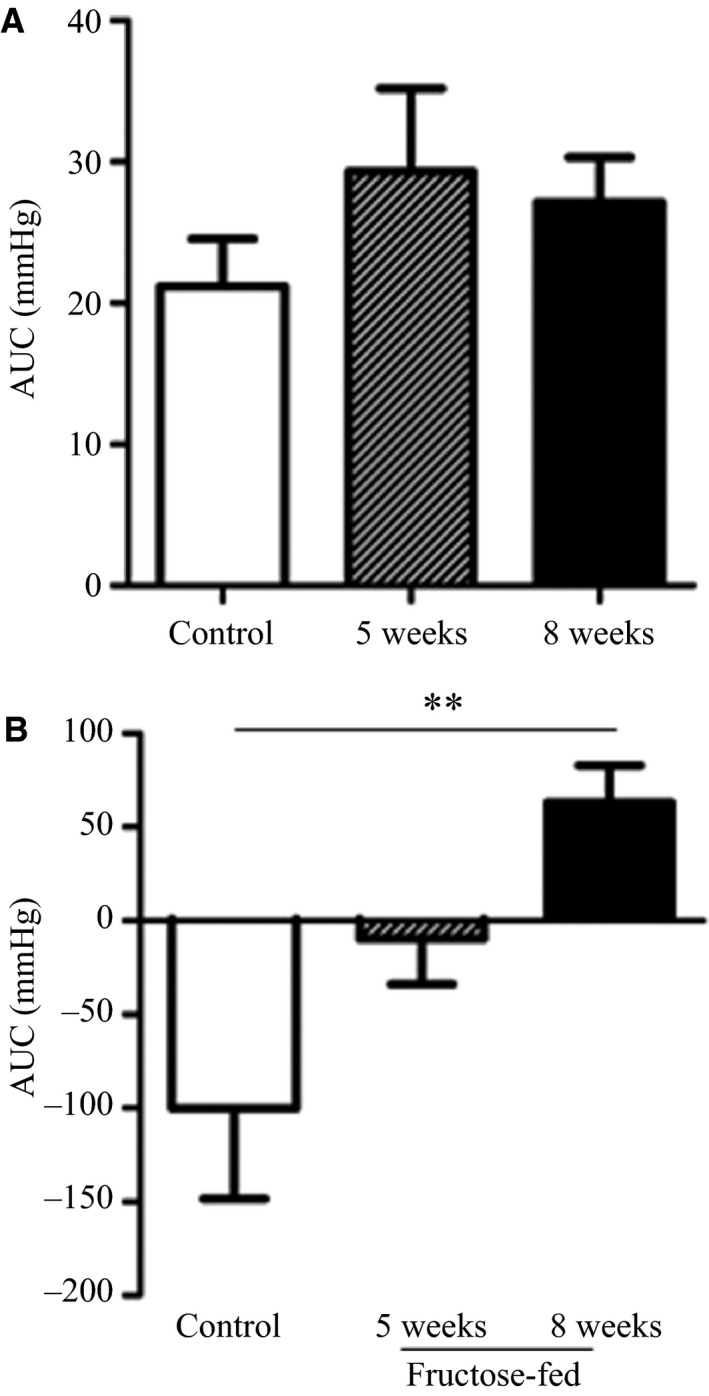
Area under the curve (AUC) during the 3‐min stress period (A) and the 20‐min recovery period (B) in control and 5‐ or 8‐week fructose‐fed rats. *n* = 7; **P < 0.01

Pressor responses to air‐jet stress were measured after 5 and 8 weeks of high‐fructose feeding. There were no differences in baseline MAP measured during in‐cage acclimation or during the 3‐min prestress period in conscious rats fed a high‐fructose diet for 5 or 8 weeks compared to control. Figure 2A shows MAP values obtained during the 3‐min prestress baseline and the 3‐min air‐jet stress period. There was an initial peak in arterial pressure with delivery of the first air pulse followed by a plateau starting 90 sec into the stress period. During the last minute of the stress period, MAP was elevated approximately 10 mmHg above prestress levels in both control and fructose‐fed animals. MAP values recorded during the 20‐min recovery period are shown in Figure 2B. At the end of recovery, MAP dropped approximately 10 mmHg below pre‐air jet baseline values in control animals, but remained higher than pre‐air jet baseline in rats fed a high‐fructose diet for 8 weeks. There was no difference in AUC across the three groups for the air‐jet stress period, while there was a marked increase in AUC during recovery in rats fed a high‐fructose diet for 8 weeks compared to control (Fig. 3).

### Blood flow responses to isoproterenol

**Figure 4:**
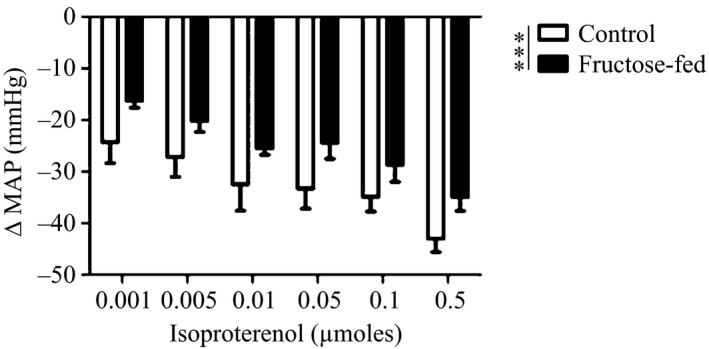
Effect of intravenous administration of isoproterenol (0.001–0.5 *μ*mol) on telemetry measurements of MAP in anesthetized control and 8‐week fructose‐fed rats. *n* = 6; ***P < 0.001 for treatment group as a source of variation in 2‐way ANOVA.

Hemodynamic responses to *β*‐adrenergic agonist isoproterenol were examined in anesthetized animals after 8 weeks of high‐fructose feeding to determine the extent to which *β*‐adrenergic responsiveness is impaired. There were no differences in baseline blood flow or vascular resistance in the renal, hind limb and mesenteric circulations (Table 2). MAP decreased in a dose‐dependent manner with isoproterenol treatment in control, but decreased to a lesser degree in fructose‐fed animals: dose (*P* < 0.001) and diet (*P* < 0.001) were significant sources of variation in two‐way ANOVA (Fig. 4). As well, the peak change in MAP with isoproterenol treatment was significantly less in the fructose‐fed mice compared to control (*P* < 0.5). There was also a dose‐dependent increase in vascular conductance after isoproterenol treatment in the hind limb and mesenteric circulations in control animals that was less pronounced in 8‐week fructose‐fed animals. In two‐way ANOVA, both dose and diet were significant sources of variation in responses to isoproterenol treatment in both the hind limb (dose: *P* < 0.001; diet: *P* < 0.01) and mesenteric vascular beds (dose: *P* < 0.05; diet: *P* < 0.01).

**Table 2:** Baseline cardiovascular parameters under anesthesia

Parameter	Control	Fructose‐fed
MAP (mmHg)	92.60 ± 3.76	102.0 ± 2.8
Heart rate (bpm)	318 ± 17.0	324 ± 4.7
Renal blood flow (mL/min/g)	5.5 ± 0.42	5.4 ± 0.35
Renal vascular resistance (mmHg·min/L)	0.05 ± 0.004	0.04 ± 0.003
Hand limb blood flow (mL/min/g)	0.1 ± 0.01	0.1 ± 0.01
Hand limb vascular resistance (mmHg·min/g)	823 ± 57.2	1084 ± 84.1
Mesenteric blood flow (mL/min/g)	1.1 ± 0.06	0.9 ± 0.06
Mesenteric vascular resistance (mmHg·min/L)	88.2 ± 10.09	112.2 ± 6.89

**Figure 5:**
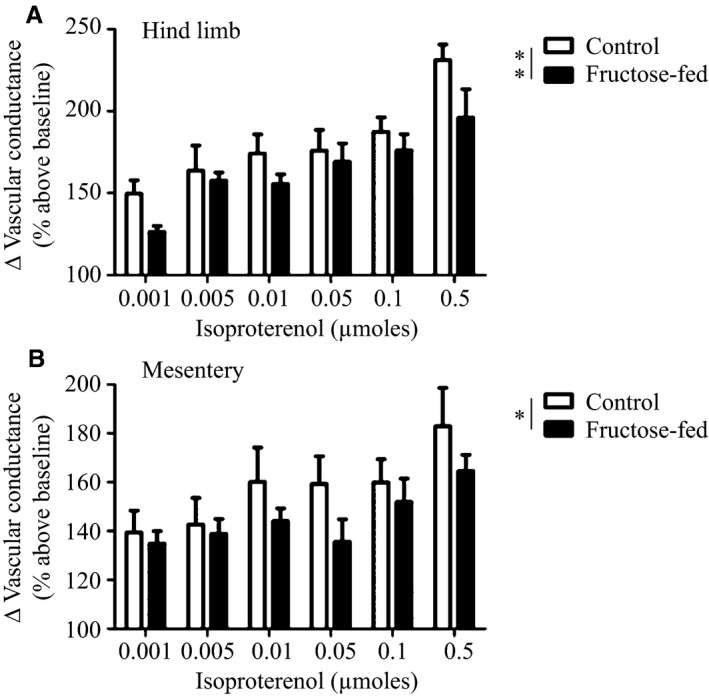
Effect of intravenous administration of isoproterenol (0.001–0.5 *μ*mol) on vascular conductance, calculated as blood flow (mL/min/g) divided by MAP (mmHg), in the hind limb (A) and mesenteric (B) vascular beds in control and 8‐week fructose‐fed rats. *n* = 6; *P < 0.5, **P < 0.01 for treatment group as a source of variation in two‐way ANOVA.

## Discussion

Prevailing thought holds that insulin resistance is central to the pathogenesis of accompanying MetS components. In accord with this view, a direct link between insulin resistance and hypertension has been reported by a substantial number of studies utilizing short‐term high‐fructose feeding to dissociate insulin resistance from obesity (Dai and McNeill 1995; Cosenzi et al. 1999; Verma et al. 1999; Galipeau et al. 2001; Katovich et al. 2001; Catena et al. 2003; Hsieh 2004). A caveat of these findings is the use of tail‐cuff plethysmography for the measurement of arterial blood pressure, which causes significant psychological stress in the animal. However, fructose‐feeding does not produce hypertension when arterial pressure is measured with telemetry, the gold standard technique for physiological blood pressure measurement. This finding suggests that the true vascular insult consequent to short‐term insulin resistance is not hypertension per se, but changes in pressor responses to acute stressful stimuli.

Short‐term fructose feeding leads to abnormalities in metabolic function characteristic of MetS, without significant changes in body weight, and thus allows for the study of events involved in the progression of cardiovascular disease. For this study, we fed LZR rats a 66% fructose diet for 8 weeks, a prolonged intervention relative to the majority of fructose‐feeding studies cited above. We previously reported this duration of fructose feeding to result in moderate insulin resistance along with hypertriglyceridemia and hypercholesterolemia (D'Angelo et al. 2005). Abnormal metabolic regulation in fructose‐fed LZR rats of the present study was evident by increased levels of fasting plasma glucose, insulin, and lipids. In the face of this metabolic dysfunction, baseline MAP in rats chronically instrumented with telemetry devices was comparable to control. This finding substantiates our previous study, which revealed unaltered 24‐h MAP measured by telemetry in Sprague–Dawley rats with fructose‐induced insulin resistance (D'Angelo et al. 2005). It is important to note that the strain and age of the animals at which the diet is initiated may influence hemodynamic responses to fructose. Three studies have reported increased MAP measured with indwelling catheters in Sprague–Dawley rats fed a high‐fructose diet for 2–5 weeks (Katakam et al. 1998, 2000a,b). However, one of these studies used young 6‐week‐old animals and all studies measured MAP 24 h after catheters were surgically placed. In contrast, the current study reports 24 h MAP recorded several weeks after telemetry placement in animals that remained housed in a familiar environment. These methodological differences may account for the discrepancy in findings with respect to baseline MAP after short‐term fructose feeding. Our results suggest that early metabolic abnormalities associated with short‐term fructose feeding do not lead to overt changes in chronic blood pressure regulation in the Zucker rat strain, consistent with our results observed previously in Sprague–Dawley rats.

In order to probe our theory that a heightened pressor response to acute stress is the functional cardiovascular manifestation of short‐term fructose feeding, we subjected animals to air‐jet stress at 5 and 8 weeks of feeding. In both fructose‐fed and control animals, MAP rapidly increased at the onset of air‐jet stress and remained higher than baseline levels throughout the 3‐min stress period. The pressor response to a fear‐eliciting stimulus is known to be mediated by mass sympathetic discharge and consequent release of catecholamines from postsynaptic adrenergic fibers and the adrenal medulla, which in turn increase heart rate and total peripheral resistance through stimulation of adrenergic receptors in the heart and blood vessels. Results of the present study show that while the magnitude of pressor responses during the 3‐min stress period was not different between groups, recovery of blood pressure was delayed in rats fed a high‐fructose diet for 8‐weeks. In control rats, MAP returned to prestress levels by 5 min into recovery and dropped approximately 10 mmHg below prestress levels by the end of the 20‐min recovery period. In contrast, MAP remained on average 10 mmHg above prestress levels throughout the 20‐min recovery period in 8‐week fructose‐fed rats.

Given that propranolol abrogates recovery following periods of mental stress (D'Angelo et al. 2006), we suspected reduced sensitivity of *β*_2_ receptors to be the primary mechanism underlying delayed poststress recovery in fructose‐fed rats. This possibility was explored by measuring MAP and regional blood flow responses to isoproterenol, a *β*‐adrenergic agonist. In animals fed a high‐fructose diet for 8 weeks, isoproterenol injection produced a less pronounced drop in MAP. Likewise, the increased conductance in both the hind limb and mesenteric circulations in response to isoproterenol was attenuated in fructose‐fed rats. Together, these results suggest that impaired blood pressure recovery after high‐fructose feeding unmasks blunted *β*_2_‐adrenergic signaling.

Several studies have demonstrated impaired *β*‐adrenergic‐mediated relaxation in prehypertensive and hypertensive patients (Feldman 1990; Stein et al. 1995; Schlaich et al. 2004) as well as in animal models of hypertension (Mallem et al. 2005). Further, targeted genetic deletion of *β*_2_ adrenergic receptors in mice results in exaggerated pressor responses to exercise and impaired isoproterenol‐induced vasodilation, without disrupting normal resting blood pressure (Chruscinski et al. 1999). Therefore, attenuated responses to isoproterenol and the associated delay in poststress blood pressure recovery observed in fructose‐fed rats of the current study may uncover early vascular defects that precede the onset of hypertension. In support of this, there exists evidence to suggest that impaired blood pressure recovery to acute psychological stress predicts future hypertension in humans (Mathews et al. 2003; Steptoe and Marmot 2003).

Endothelial dysfunction, a hallmark of the prehypertensive state, may be wholly or partly responsible for blunted *β*‐adrenergic‐mediated relaxation in fructose‐fed rats. Previous studies have demonstrated attenuation of isoproterenol‐induced relaxation after removal of the endothelium or inhibition of nitric oxide synthesis by L‐NAME (Cardillo et al. 1997; Majmudar et al. 1999; Mallem et al. 2005), suggesting that endothelial production of nitric oxide (NO) contributes to *β*_2_‐adrenergic relaxation. Given our published findings demonstrating blunted endothelium‐dependent relaxation in LZR rats fed a high‐fructose diet for 8 weeks (Romanko et al. 2009), it is possible that endothelial dysfunction contributes to impaired responses to *β*_2_ stimulation and delayed poststress recovery of blood pressure in fructose‐fed rats of this study. A study by Schlaich et al., reported that inhibition of eNOS blunted *β*‐adrenergic‐mediated dilation in normotensive subjects, but not in their hypertensive and prehypertensive counterparts (Ferro et al. 2004), thus implicating deficient NO‐induced *β*_2_ adrenergic relaxation as an early event in the pathogenesis of hypertension.

Prolonged sympathoexcitation represents another possible contributor to impaired blood pressure recovery to acute mental stress in fructose‐fed rats. Hyperactivity of the sympathetic nervous system is a common feature of MetS and plays an important role in the pathogenesis of hypertension (Straznicky et al. 2014). Studies in spontaneously hypertensive rats demonstrate that increased sympathetic output predates the onset of hypertension (Simms et al. 2009). Moreover, there have been a number of studies reporting a causative link between autonomic imbalance and insulin resistance, most of which demonstrate the sympathoexcitatory effects of insulin (Rahmouni et al. 2004). Interestingly, sympathetic responses to a cold‐pressor test were shown to predict future insulin resistance in men (Flaa et al. 2008) and autonomic dysfunction preceded the onset of metabolic dysfunction in a mouse model of fructose overload (De Angelis et al. 2012). Therefore, it is possible that along with blunted *β*_2_ responsiveness, persistent sympathetic activation during the poststress period contributes to delayed blood pressure recovery in fructose‐fed rats of this study.

### Perspectives and significance

We show herein that early cardiovascular effects of metabolic dysfunction consequent to short‐term high‐fructose feeding manifest as delayed blood pressure recovery to acute mental stress. Further, our data provide evidence to support a role for reduced *β*_2_‐adrenergic‐mediated vascular relaxation in this fructose‐induced impairment in acute blood pressure control. Together, these findings highlight impaired *β*_2_‐adrenergic responsiveness, reflected in delayed blood pressure recovery to adrenergic stimulation, as an important antecedent to hypertension in MetS.

## Conflicts of Interest

None declared.
